# Workplace bullying in a German representative sample of employees: a cross-sectional analysis

**DOI:** 10.1186/s12995-026-00535-3

**Published:** 2026-10-21

**Authors:** Franziska D. Welzel, Franziska Ulrike Jung, Alexander Pabst, Antonia M. Buß, Hermann Burr, Uwe Rose, Andreas Seidler, Steffi G. Riedel-Heller, Margrit Löbner

**Affiliations:** 1 Institute of Social Medicine, Occupational Health and Public Health, Medical Faculty, University of Leipzig, Philipp-Rosenthal-Straße 55, 04103 Leipzig, Germany; 2 Federal Institute for Occupational Safety and Health (BAuA), Berlin, Germany; 3 Institute and Policlinic of Occupational and Social Medicine (IPAS), Faculty of Medicine, TU Dresden, Dresden, Germany

**Keywords:** Adult, Employment, Occupation, Prevalence, Work behavior

## Abstract

**Background:**

Workplace bullying (WB) is linked to a variety of detrimental health outcomes among affected individuals, while also imposing negative organizational consequences on the businesses involved. The objective of the present study was to update prevalences of WB and to investigate associations with WB distinguished by the source of bullying (from co-workers, supervisors, or both) in a representative sample of employees in Germany.

**Methods:**

The study is based on a representative cross-sectional telephone survey comprising a sample of *N* = 5,015 employed adults. WB was defined as cases in which respondents indicated that they had experienced unfair criticism, harassment, or public humiliation by co-workers and/or supervisors on a weekly basis over the previous six months. To identify associations of WB, multivariable logistic regression analyses were run, distinguished by the source of bullying.

**Results:**

The prevalence of WB was 6.5% overall, with 4.4% reporting WB by co-workers and 3.5% reporting WB by supervisors. WB decreased with age, with the highest prevalence among young adults aged 18 to 29 years (11.5%). Individuals in an apprentice status (12.3%), or those working in blue-collar jobs (10.2%) showed the highest prevalence for WB. In multivariable analyses, only age was significantly associated with WB across all sources of WB showing a higher vulnerability for younger employees. WB by co-workers and WB by supervisors were strongly linked.

**Conclusions:**

One in fifteen employed individuals experiences WB in Germany. Young age was the most consistent factor associated with WB. Partial associations of some variables (gender, region, professional status) highlight the context-dependent nature of WB. Furthermore, the strong link between WB by co-workers and WB by supervisors underscores the systemic nature of this work phenomenon. Implementing preventive strategies that specifically address WB is needed to sustain employee health.

**Supplementary Information:**

The online version contains supplementary material available at https://doi.org/10.1186/s12995-026-00535-3.

## Background

Workplace bullying (WB) is described as repeated and hostile or excluding behavior towards another person at work exploiting or pushing the victim in an inferior position and towards feelings of helplessness [1, 2]. To differentiate bullying from other uncivil or aggressive behaviors, such as harassment, violence or conflicts, the frequency as well as the duration of negative behaviors are important. According to Leymann [1] the definition of WB requires hostile actions to occur on a weekly basis for at least six months. While WB can occur in any direction, bullying against co-workers and bullying against subordinate employees have been described most frequently [3, 4].

The prevalence of WB varies considerably depending on methodological and geographical factors [5]. A meta-analysis including samples from 24 countries and one multinational sample estimated the overall prevalence of WB at 14.6% [5]. Other studies report that up to 30% of employees experience bullying in the workplace sometime during their working life [6, 7]. For Germany, studies using representative samples reported prevalence estimates of 5.5% to 6.7% [8, 9].

Generally, it appears that certain groups are particularly vulnerable to experiencing WB. Some studies found that people with a different cultural background, unmarried individuals, as well as female or younger workers are at a higher risk of being bullied at work [9–11]. With regard to differences in occupations and economic sectors, research seems to indicate higher risks for the occurrence of WB in the health care sector [12]. However, comparative studies of WB across occupational areas have rarely been conducted. A cross-sectional study assessing WB among the working population in southeastern France identified an increased risk among men employed in lower-tier white- and blue-collar occupations and associate professional roles [13]. For women, the highest risk was associated with positions as government associate professionals [13]. Generally, it is still not well understood in which occupational area WB is specifically prevalent and if the source of bullying (top-down vs. lateral) plays a role in the occurrence of this high impact work phenomenon.

Bullying is described as one of the main causes of stress in the workplace and has frequently been linked to detrimental effects on both the individuals and the organizations involved [14–16]. Bullied individuals report poorer levels of job satisfaction, life satisfaction, and well-being [14–16]. Consequently, individuals affected by WB report poorer performance at work, and longer or more frequent sick leave, which in turn may impact the businesses involved, as these factors can result in decreased productivity and absenteeism [15, 17, 18]. As a result, WB is associated with high economic costs [8].

Although WB has gained attention as a complex work phenomenon in the more recent research literature, several research gaps remain. For Germany, representative data on the prevalence of WB is over a decade old and therefore does not account for the impactful changes on organizations and work environments within the last five to ten years (e.g., such as an increase in remote work). Additionally, the specific risk factors associated with WB may differ based on who the actors are, highlighting the importance of distinguishing between bullying by supervisors and co-workers. To the best of our knowledge, a differentiated analysis of factors associated with WB based on the source of bullying is missing (so far).

The aim of the present study is to estimate current prevalences of WB in a representative sample of employees. The study further aims to assess how sociodemographic and occupational factors are related to WB differentiated by the source of bullying (WB by co-workers vs. WB by supervisors vs. WB by co-workers and/or supervisors).

## Methods

### Study design and context information

This study is part of the project, “Workplace bullying among the working population in Germany – a representative study,“ which was conducted by the Institute of Social Medicine, Occupational Medicine and Public Health (ISAP) at Leipzig University on behalf of the German Federal Ministry of Labour and Social Affairs. The study comprises a cross-sectional telephone survey representative of employed adults in Germany. Detailed information on the study procedure have been published previously [19].

### Inclusion/exclusion criteria

Inclusion criteria were: (a) age 18 + years, (b) sufficient proficiency in the German language, (c) being employed (including blue-collar workers, salaried employees, apprentices, civil servants and marginally employed individuals), (d) having paid employment for at least one hour per week.

Exclusion criteria were: (a) self-employment; (b) freelancing; (c) working as a family member’s helper; (d) being a student; and (e) being unemployed or currently experiencing an interruption in employment (for example due to parental leave, caregiver leave).

### Sample and survey procedure

**Fig. 1:**
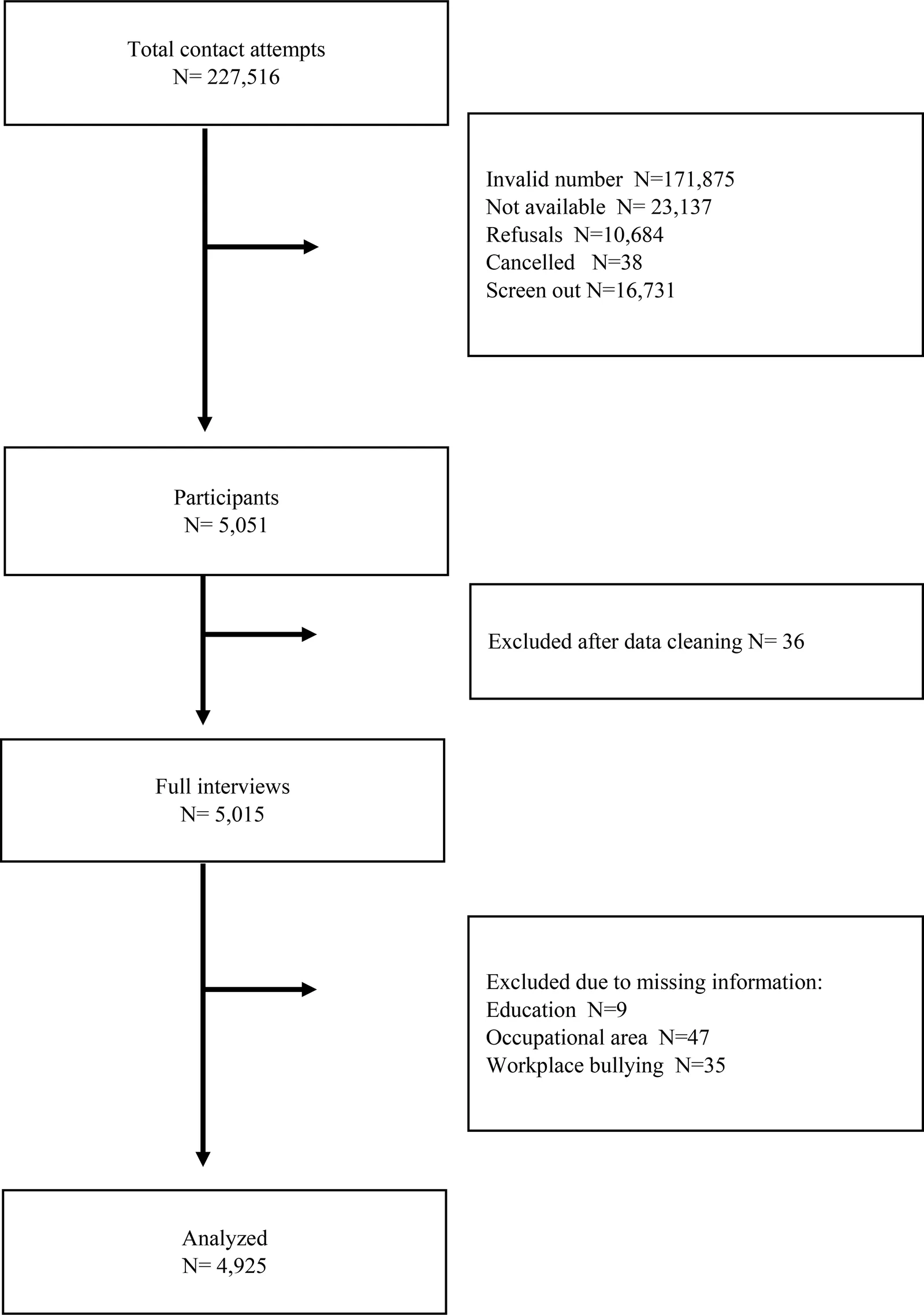
Flowchart of the study sample

The survey comprised standardized computer-assisted telephone interviews conducted by the market and social research institute USUMA GmbH. Data collection took place from August 2023 to January 2024. The survey followed a sampling design established by the Working Group of German Market and Social Research Institutes (ADM), which allows for a controlled sample selection based on regional and demographic parameters. A dual-frame technique was used, which includes landline and mobile phone numbers. To contact participants via landline, registered and generated phone numbers were drawn from the ADM telephone sample “EASYSAMPLE” using the Federal Network Agency’s area network system across Germany, stratified by the known location size classes of the individual districts or municipalities, and proportionate to the regional population structure at the federal state level. This procedure allows for a random selection of households. For households contacted via landline, when more than one individual aged 18 or older was in dependent employment, the respondent within the selected household was randomly chosen using the Kish Selection Grid [20]. Through applying this selection method, eligible individuals within a selected household had an equal probability of participation. Given that mobile phones are linked to individuals rather than specific households, no within-household selection method was applied for targets contacted via mobile phone numbers. Mobile phone numbers were chosen from the ADM sample “EASYMOBILE”. The share of mobile phone numbers in the sample was 41%. In total, 227,516 contact attempts were made during the survey. Of these, N = 222,465 did not lead to a completed interview. Reasons were invalid numbers (N = 171,875), individuals being unavailable (e.g., answering machine; N = 23,137), refusals to take part in the interview (N = 10,684), interviews prematurely terminated by individuals (N = 38), and individuals not meeting the inclusion or exclusion criteria (N = 16,731). In total, N = 5,051 participants were interviewed, corresponding to a response of 19.1%, calculated according to the standards of the American Association for Public Opinion Research (AAPOR, 2020; [21]). A detailed description of the analysis of the response rate has been published in the corresponding methods report [22]. Data cleaning was conducted by USUMA and included reviewing the data for correct application of questionnaire filters, and logical and internally consistent responses (e.g., regarding the reporting of employment status). After data cleaning, the final study sample comprised N = 5,015 participants. After exclusion of individuals with missing data on relevant variables a sample of N = 4,925 was included in the analyses. The sample selection is shown in Fig. 1.

### Outcomes and measures

#### Workplace bullying

WB was assessed using items adapted from the “Study on Mental Health at Work” [9, 23]. Participants were asked, “Do you often feel unfairly criticized, harassed, or embarrassed in front of others by co-workers?” Those who responded “yes” were asked how often they had been the target of these actions over the previous six months (“daily, at least once a week, at least once a month, less than once a month”). The same questions were asked regarding the occurrence and frequency of negative actions demonstrated by supervisors. Based on those items, overall WB was operationalized as follows: the person felt unfairly criticized, harassed or embarrassed in front of others by co-workers and/or supervisors and such incidents occurred “daily” or “at least once a week” during the previous six months [22]. WB by co-workers (supervisors) was defined as present when a person felt unfairly criticized, harassed or embarrassed in front of others by co-workers (supervisors) and such incidents occurred at the same frequency (“daily” or “at least once a week”) within the same time frame (during the previous six months) [22]. Thus, overall WB refers to any bullying experience involving supervisors, co-workers, or both, whereas WB by co-workers (or supervisors) refers to bullying from that source, irrespective of whether the other source was also involved. Indicators of WB were operationalized as binary variables.

#### Sociodemographics and occupational area

Sociodemographics included age, gender, education, professional status and geographic region. Educational status was assessed using the CASMIN classification [24] which combines information on school and vocational qualification. Region was assessed at the federal state level and categorized into: West (North Rhine–Westphalia, Hesse, Saarland, and Rhineland-Palatinate), East (Mecklenburg–Western Pomerania, Brandenburg, Berlin, Saxony-Anhalt, Thuringia, and Saxony), North (Schleswig–Holstein, Lower Saxony, Hamburg, and Bremen), and South (Bavaria, Baden-Württemberg). Professional status categories were based on self-report and distinguished, in accordance with the 2023 German Microcensus, into the following mutually exclusive categories: civil servants (individuals employed under public-law service relationship), apprentices (individuals in vocational training), blue-collar workers (employees classified as laborers), and salaried employees (comprises all other employees, similar to white-collar workers). Each participant was assigned to one category only [25]. Occupational area was assessed by asking “Which sector does the company/organization you work for belong to?” using standardized response categories derived from the classification of occupations issued by the German Federal Employment Agency [26]. In cases where participants could not identify within the predefined categories and selected “other,” (*n* = 1058) the occupational area was determined using responses to three occupation-related questions: current occupation, a detailed description of duties, and any alternative job titles.

### Statistical analysis

Weighting procedures were implemented to achieve representativeness of the sample with respect to the general population of employed residents in Germany. First, a design weighting was conducted to account for differing selection probabilities among target individuals. Design weighting was performed using the standard calculation procedure for the dual-frame design recommended by the ADM [27]. The design weighting corrects for a higher selection probability of target persons in smaller households. Subsequently, an adjustment weighting was implemented to mitigate potential biases resulting from nonresponse at the individual level and to align the sample with the known population distributions of relevant characteristics. A detailed description of the applied weighting procedure have been published previously [22]. Weighting accounted for age, gender and their combinations, federal state, household size, and professional status group (apprentice, civil servant, blue-collar worker, and salaried employee) based on the 2023 census data [25].

Results are presented as weighted estimations; only number of cases are presented unweighted. Descriptive statistics are presented as means with SD and range (age), and number of cases with percentages (qualitative measures). Differences between univariate characteristics and overall WB were tested using logistic regression analysis (age as independent variable) and ꭓ2 tests (for categorical variables).

Prevalence estimates of overall WB, WB by supervisors and WB by co-workers across sociodemographics are presented as numbers of cases and percentages with 95% confidence intervals (CI). For illustrative purposes, age in years was grouped into five categories (18–29, 30–39, 40–49, 50–59, and ≥ 60).

Multivariable logistic regression analyses were run to identify associations of WB. Three separate regression analyses were run distinguished by the source of WB (overall WB, WB by co-workers, and WB by supervisors). All analyses included continuous age as well as indicators of gender, education, professional status, region, and occupational area as covariates. Analysis for WB by co-workers (WB by supervisors) additionally accounted for the occurrence of WB by supervisors (co-workers) in order to estimate associations between predictor variables and WB by the specific perpetrator group independent of the exposure to WB by the other perpetrator group. Multivariable analyses of WB were re-estimated to include interaction terms, allowing for the assessment of potential moderating relationships of age with gender, professional status, and occupational area, and of gender with professional status, and occupational area. Results are presented as odds ratios (OR) and their corresponding 95% CI. For region, professional status, and occupational area effect coding was used to compare each category’s effect to the overall mean. Wald-tests are presented for multinomial variables. In all regression analyses, robust standard errors were estimated. Statistical analyses were carried out using STATA 16.1 SE (StataCorp LLC). The level of statistical significance was set to *p* < 0.05 for all analyses.

### Nonresponse analyses

Cases with incomplete data (*n* = 90, 1.8%) were excluded from the analyses. Individuals excluded from the analyses did not significantly differ from the study sample in terms of age (excluded vs. included: M = 43.2, SD = 1.9 vs. M = 42.7, SD = 0.3; *p* = 0.782) or gender (excluded vs. included: 40.1% female vs. 48.2% female; ꭓ^2^_(1)_ = 1.4489, *p* = 0.228). However, excluded individuals more often had a lower educational level (excluded vs. included: 24.9% vs. 8.3% low education, 48.4% vs. 52.4% medium education, 26.7% vs. 39.3% high education; ꭓ^2^_(2)_ = 7.7672, *p* = 0.0008).

## Results

### Sample characteristics

Table 1 displays the main characteristics of the study sample. The sample was on average 42.7 years old (SD = 0.3). About half of the sample was male (51.8%) and had a medium level of education (52.4%). Individuals affected by WB were younger (36.9 vs. 43.1), had more often an apprentice status (7.4% vs. 3.7%) or worked in blue-collar jobs (19.6% vs. 11.9%), compared to those who had not experienced WB. In terms of occupational area, a higher proportion of those affected by WB worked in the construction sector (including occupations in construction, architecture, surveying and technical building services), as compared to those not affected by WB (13.2% vs. 7.4%). The average working time in the sample was 36.5 h per week.

**Table 1:** Sociodemographic and workplace characteristics of the total sample and by experience of workplace bullying (WB)

	total sample, *N* = 4,925		WB, *N* = 267		no WB, *N* = 4,658	
	mean(sd)	*n* (%)	mean(sd)	*n* (%)	mean(sd)	*n* (%)
**Age**	42.7(0.3)		36.9(0.9)		43.1(0.3)	
**G** **ender**						
Male		2,367 (51.8)		122 (47.4)		2,245 (52.1)
Female		2,558 (48.2)		145 (52.5)		2,413 (47.9)
**E** **ducation** ^**b**^						
High		2,106 (39.3)		97 (31.8)		2,009 (39.8)
Middle		2,421 (52.4)		144 (58.9)		2,277 (51.9)
Low		398 (8.3)		26 (9.3)		372 (8.2)
**R** **egion**						
North		829 (16.1)		46 (18.8)		783 (15.9)
West		1,626 (34.3)		83 (32.0)		1,543 (34.5)
South		1,456 (30.9)		95 (36.9)		1,361 (30.5)
East		1,014 (18.7)		43 (12.2)		971 (19.1)
**P** **rofessional status**						
Apprentice		52 (3.9)		8 (7.4)		44 (3.7)
Salaried employee		3,994 (78.1)		203 (69.6)		3,791 (78.7)
Civil servant		395 (5.5)		15 (3.4)		380 (5.6)
Blue-collar worker		484 (12.4)		41 (19.6)		443 (11.9)
**O** **ccupational area**						
Occupations in agriculture, forestry, farming, and landscaping		85 (1.8)		4 (0.6)		81 (1.9)
Occupations in production of raw materials and goods, and manufacturing		686 (15.2)		40 (17.3)		646 (15.1)
Occupations in construction, architecture, surveying and technical building services		374 (7.8)		31 (13.2)		343 (7.4)
Occupations in natural sciences, geography and informatics		297 (6.2)		12 (2.8)		285 (6.4)
Occupations in transit, logistics, safety and security, and military		438 (9.7)		31 (11.9)		407 (9.6)
Occupations in commercial services, trading, sales, the hotel business and tourism		731 (14.9)		39 (15.9)		692 (14.9)
Occupations in business organization, accounting, law and administration		638 (11.6)		20 (6.4)		618 (11.9)
Occupations in health care, the social sector, teaching and education		1,465 (28.4)		80 (28.7)		1,385 (28.3)
Occupations in philology, literature, humanities, social sciences, economics, media, art, culture, and design		211 (4.3)		10 (2.9)		201 (4.4)

Note: WB = workplace bullying; N = unweighted total sample size; n = unweighted sample size of subgroups; % = weighted percentage; SD = standard deviation; CI = confidence interval; ^a^variables analysed based on chi-squared tests or logistic regression, as appropriate; ^b^classification according to the international new CASMIN educational classification [24]

### Prevalences of workplace bullying

The prevalences of WB are presented in Table 2. The prevalence of overall WB was 6.5% (95%-CI 5.6–7.5) in the total sample. WB by co-workers was slightly more prevalent (4.4%) than WB by supervisors (3.5%). A proportion of 1.4% (*N* = 65) of the sample experienced both WB by co-workers and supervisors. With regard to age, overall WB was most frequently reported among individuals aged 18 to 29 years (11.5%), with prevalences declining across older age groups. This age-related pattern was consistent across all forms of WB, whether experienced by co-workers or by supervisors. Prevalences of WB were slightly higher for women than men (overall WB: 7.1% vs. 5.9%, WB by co-workers: 5.0% vs. 3.9%, WB by supervisors: 3.8% vs. 3.2%). Similarly, individuals with lower educational attainment more frequently reported overall WB (low: 7.3%; medium: 7.3%; high: 5.2%) as well as WB by co-workers (low: 7.4%; medium: 4.6%; high: 3.6%). WB by supervisors was more prevalent among individuals with an intermediate level of education. Furthermore, overall WB was most prevalent among individuals with an apprentice status (12.3%), or blue-collar workers (10.2%). Although this pattern was apparent across both sources of WB, it appeared to be less pronounced among WB by supervisors. Regarding the occupational area, overall WB was most prevalent among occupations in construction, architecture, surveying and technical building services (11.1%). A similar pattern can be observed for WB by co-workers (see Table 3). WB by supervisors was most prevalent among occupations in transportation (including transit, logistics, safety and security, and military; 4.9%) followed by occupations in manufacturing (including production of raw materials and goods, and manufacturing; 4.8%). In addition, sensitivity analyses were conducted among the subsample of individuals with 25 or more working hours per week (*N* = 4,311). Prevalence estimates among the subsample are similar to the overall sample showing no severe distortion due to individuals with minimal weekly working hours. Prevalence estimates of the subsample are presented in the supplement (Table S1).

**Table 2:** Prevalences of workplace bullying (WB) by source

	WB by supervisors/co-workers			WB by co-workers			WB by supervisors		
	*n*/*N*	%	[95%CI]	*n*/*N*	%	[95%CI]	*n*/*N*	%	95%CI
**T** **otal**	267/4,925	6.5	[5.6–7.5]	179/4,851	4.4	[3.7–5.3]	153/4,834	3.5	[2.9–4.3]
**Age**									
18–29	45/350	11.5	[8.4–15.5]	33/350	7.9	[5.5–11.4]	24/343	5.9	[3.7–9.1]
30–39	58/714	7.2	[5.5–9.4]	40/705	5.1	[3.7–7.1]	30/701	3.6	[2.4–5.2]
40–49	69/1,052	6.5	[4.9–8.4]	49/1,047	4.4	[3.2–5.9]	41/1,039	3.9	[2.7–5.5]
50–59	61/1,884	3.2	[2.4–4.2]	35/1,851	1.9	[1.3–2.9]	38/1,850	1.9	[1.3–2.6]
60+	34/925	3.3	[2.2–4.7]	22/898	2.0	[1.2–3.2]	20/901	2.0	[1.2–3.2]
**G** **ender**									
Male	122/2,367	5.9	[4.8–7.3]	80/2,342	3.9	[3.0-5.1]	70/2,320	3.2	[2.4–4.3]
Female	145/2,558	7.1	[5.7–8.7]	99/2,509	5.0	[3.9–6.4]	83/2,514	3.8	[2.8–5.1]
**E** **ducation** ^**a**^									
High	97/2,106	5.2	[4.1–6.6]	62/2,086	3.6	[2.7–4.8]	56/2,049	2.9	[2.1–3.9]
Middle	144/2,421	7.3	[5.9–8.9]	92/2,383	4.6	[3.5–5.9]	84/2,391	4.1	[3.1–5.4]
Low	26/398	7.3	[4.5–11.6]	25/382	7.4	[4.5–11.8]	13/394	2.8	[1.4–5.2]
**Region**									
North	46/829	7.6	[5.2–10.9]	29/811	4.4	[2.7-7.0]	30/809	4.9	[3.0-7.7]
West	83/1,626	6.1	[4.6–7.9]	58/1,602	4.6	[3.3–6.4]	48/1,597	3.1	[2.1–4.4]
South	95/1,456	7.8	[6.1–9.9]	62/1,437	4.8	[3.5–6.5]	49/1,430	4.2	[3.0-5.9]
East	43/1,014	4.2	[2.9–6.1]	30/1,001	3.4	[2.2–5.2]	26/998	1.9	[1.2-3.0]
**P** **rofessional status**									
Apprentice	8/52	12.3	[5.7–24.3]	6/52	9.2	[3.9–20.1]	3/51	4.4	[1.2–15.2]
Salaried employee	203/3,994	5.8	[4.9–6.8]	133/3,932	3.8	[3.1–4.6]	119/3,920	3.4	[2.7–4.2]
Civil servant	15/395	4.0	[2.1–7.5]	11/392	2.8	[1.4–5.7]	9/386	2.8	[1.2–6.4]
Blue-collar worker	41/484	10.2	[7.1–14.5]	29/475	7.7	[5.0-11.6]	22/477	4.4	[2.6–7.4]
**O** **ccupational area**									
Occupations in agriculture, forestry, farming, and landscaping	4/85	2.1	[0.7–6.1]	3/83	1.6	[0.4–5.7]	4/83	2.1	[0.7–6.2]
Occupations in production of raw materials and goods, and manufacturing	40/686	7.4	[5.1–10.6]	25/684	4.0	[2.5–6.2]	25/680	4.8	[2.9–7.8]
Occupations in construction, architecture, surveying and technical building services	31/374	11.1	[7.3–16.4]	22/366	8.6	[5.2–13.8]	16/368	4.7	[2.6–8.1]
Occupations in natural sciences, geography and informatics	12/297	2.9	[1.5–5.7]	8/294	1.7	[0.7–3.6]	8/293	2.1	[0.9–4.8]
Occupations in transit, logistics, safety and security, and military	31/438	8.0	[5.3–12.0]	20/430	5.7	[3.4–9.3]	19/424	4.9	[2.9–8.2]
Occupations in commercial services, trading, sales, the hotel business and tourism	39/731	6.9	[4.7–10.0]	24/724	4.8	[2.9–7.7]	25/715	3.8	[2.2–6.4]
Occupations in business organization, accounting, law and administration	20/638	3.6	[2.1-6.0]	13/629	2.3	[1.2–4.5]	14/630	2.5	[1.3–4.6]
Occupations in health care, the social sector, teaching and education	80/1,465	6.6	[4.9–8.8]	58/1,433	4.8	[3.4–6.8]	37/1,436	2.8	[1.8–4.4]
Occupations in philology, literature, humanities, social sciences, economics, media, art, culture, and design	10/211	4.4	[2.1–8.8]	6/208	2.6	[1.0-6.8]	5/205	2.5	[0.9–6.2]

Note: WB = workplace bullying; N = unweighted total sample size; n = unweighted sample size of subgroups affected by WB; % = weighted percentage; SD = standard deviation; CI = confidence interval; ^a^classification according to the international new CASMIN educational classification [24]

**Table 3:** Results of the logistic regression analysis for overall workplace bullying (WB)

	WB by supervisors and/or co-workers			
	*n*/*N*	OR	[95%CI]	*p*-value
**A** **ge**		0.96	[0.95–0.97]	< 0.001
**G** **ender**				
Male	122/2,367	Ref.		
Female	145/2,558	1.51	[1.05–2.17]	0.025
**E** **ducation** ^**a**^				
Low	97/2,106	Ref.		
Middle	144/2,421	1.08	[0.60–1.93]	0.792
High	26/398	0.93	[0.48–1.78]	0.830
(WALD)		ꭓ^2^ = 0.69		0.707
**R** **egion** ^**b**^				
North	46/829	1.23	[0.90–1.70]	0.185
West	83/1,626	0.97	[0.74–1.27]	0.841
South	95/1,456	1.22	[0.95–1.58]	0.113
East	43/1,014	0.67	[0.48–0.92]	0.016
(WALD)		ꭓ^2^ = 7.28		0.063
**P** **rofessional status** ^**b**^				
Apprentice	8/52	0.89	[0.45–1.77]	0.753
Salaried employee	203/3,994	0.90	[0.65–1.26]	0.559
Civil servant	15/395	0.77	[0.42–1.38]	0.385
Blue-collar worker	41/484	1.59	[1.02–2.48]	0.037
(WALD)		ꭓ^2^ = 5.74		0.125
**O** **ccupational area** ^**b**^				
Occupations in agriculture, forestry, farming, and landscaping	4/85	0.37	[0.13–1.07]	0.066
Occupations in production of raw materials and goods, and manufacturing	40/686	1.35	[0.88–2.07]	0.163
Occupations in construction, architecture, surveying and technical building services	31/374	2.31	[1.46–3.63]	< 0.001
Occupations in natural sciences, geography and informatics	12/297	0.61	[0.32–1.18]	0.145
Occupations in transit, logistics, safety and security, and military	31/438	1.52	[0.96–2.40]	0.073
Occupations in commercial services, trading, sales, the hotel business and tourism	39/731	1.21	[0.79–1.84]	0.363
Occupations in business organization, accounting, law and administration	20/638	0.70	[0.41–1.19]	0.194
Occupations in health care, the social sector, teaching and education	80/1,465	1.19	[0.82–1.72]	0.343
Occupations in philology, literature, humanities, social sciences, economics, media, art, culture, and design	10/211	0.88	[0.43–1.82]	0.743
(WALD)		ꭓ^2^ = 20.35		0.009

Note: WB = workplace bullying; OR = odds ratio; Ref. = reference category; CI = confidence interval; ꭓ^2^ = chi-square statistic; ^a^classification according to the international new CASMIN educational classification [24]; ^b^deviation coding (effects coding) was applied, odds ratios thus reflect comparisons of each category to the overall average outcome across categories

### Associations of workplace bullying

The results of the multivariable logistic regression analyses are shown in Tables 3 and 4.

**Table 4:** Results of the logistic regression analyses for workplace bullying (WB) by co-workers and by supervisors

	WB by co-workers				WB by supervisors			
	*n*/*N*	OR	[95%CI]	*p*-value	*n*/*N*	OR	[95%CI]	*p*-value
**Age**		0.96	[0.94–0.98]	< 0.001		0.97	[0.95–0.99]	0.033
**G** **ender**								
Male	80/2,342	Ref.			70/2,320	Ref.		
Female	99/2,509	1.48	[0.92–2.36]	0.100	83/2,514	1.34	[0.77–2.34]	0.289
**E** **ducation** ^**a**^								
Low	62/2,086	Ref.			56/2,049	Ref.		
Middle	92/2,383	0.52	[0.26–1.02]	0.057	84/2,391	2.04	[0.96–4.33]	0.062
High	25/382	0.56	[0.26–1.20]	0.139	13/394	1.50	[0.69–3.28]	0.303
(WALD)		ꭓ^2^ = 3.61		0.164		ꭓ^2^ = 4.11		0.128
**Region** ^**b**^								
North	29/811	0.81	[0.49–1.33]	0.413	30/809	1.54	[0.97–2.44]	0.063
West	58/1,602	1.21	[0.86–1.69]	0.261	48/1,597	0.87	[0.61–1.23]	0.448
South	62/1,437	1.02	[0.70–1.47]	0.912	49/1,430	1.24	[0.85–1.80]	0.246
East	30/1,001	0.99	[0.66–1.48]	0.983	26/998	0.59	[0.39–0.88]	0.010
(WALD)		ꭓ^2^ = 1.41		0.704		ꭓ^2^ = 8.98		0.029
**P** **rofessional status** ^**b**^								
Apprentice	6/52	1.06	[0.43–2.60]	0.887	3/51	0.60	[0.16–2.14]	0.432
Salaried employee	133/3,932	0.79	[0.52–1.19]	0.268	119/3,920	1.17	[0.69-2.00]	0.550
Civil servant	11/392	0.65	[0.35–1.23]	0.193	9/386	1.42	[0.64–3.16]	0.386
Blue-collar worker	29/475	1.79	[0.97–3.28]	0.059	22/477	0.99	[0.47–2.08]	0.985
(WALD)		ꭓ^2^ = 6.09		0.107		ꭓ^2^ = 1.06		0.786
**O** **ccupational area** ^**b**^								
Occupations in agriculture, forestry, farming, and landscaping	3/83	0.43	[0.16–1.14]	0.091	4/83	0.94	[0.37–2.37]	0.908
Occupations in production of raw materials and goods, and manufacturing	25/684	0.89	[0.49–1.62]	0.712	25/680	1.68	[0.94-3.00]	0.077
Occupations in construction, architecture, surveying and technical building services	22/366	2.38	[1.31–4.31]	0.004	16/368	1.13	[0.56–2.27]	0.721
Occupations in natural sciences, geography and informatics	8/294	0.57	[0.27–1.21]	0.148	8/293	0.83	[0.37–1.84]	0.649
Occupations in transit, logistics, safety and security, and military	20/430	1.51	[0.85–2.69]	0.152	19/424	1.34	[0.73–2.46]	0.337
Occupations in commercial services, trading, sales, the hotel business and tourism	24/724	1.21	[0.72–2.04]	0.464	25/715	1.02	[0.60–1.74]	0.920
Occupations in business organization, accounting, law and administration	13/629	0.78	[0.41–1.49]	0.468	14/630	0.80	[0.42–1.49]	0.488
Occupations in health care, the social sector, teaching and education	58/1,433	1.48	[0.92–2.36]	0.097	37/1,436	0.70	[0.39–1.26]	0.244
Occupations in philology, literature, humanities, social sciences, economics, media, art, culture, and design	6/208	0.87	[0.33–2.24]	0.776	5/205	0.84	[0.35–2.02]	0.709
(WALD)		ꭓ^2^ = 17.06		0.029		ꭓ^2^ = 4.81		0.777
**Bullied by supervisors**	65/151	19.21	[11.38–32.42]	< 0.001	-	-	-	-
**Bullied by co-workers**	-	-	-	-	65/177	18.97	[11.28–31.88]	< 0.001

Note: WB = workplace bullying; OR = odds ratio; Ref. = reference category; CI = confidence interval; ꭓ^2^ = chi-square statistic; ^a^classification according to the international new CASMIN educational classification [24]; ^b^deviation coding (effects coding) was applied, odds ratios thus reflect comparisons of each category to the overall average outcome across categories

Variables found to be significantly associated with overall WB were age, gender, and occupational area. The odds of experiencing overall WB were 4% lower with each additional year (OR: 0.96, 95% CI: 0.95–0.97). For women the odds of experiencing WB were 1.51 times larger compared to men (95% CI: 1.05–2.17). Furthermore, individuals in occupations in construction, architecture, surveying and technical building services had significantly higher odds of being bullied (OR: 2.31, 95% CI: 1.46–3.63) compared to the average odds across occupational groups. In addition, the results show lower odds of overall WB for the region of East Germany (OR: 0.67, 95% CI: 0.48–0.92) and higher odds for blue-collar workers (OR: 1.59, 95% CI: 1.02–2.48) than on average. However, the findings on region and professional status need to be interpreted with caution as the Wald tests indicated no statistically significant effects for either geographic region or professional status overall.

Regarding WB by co-workers, results show significant associations of age and occupational area with co-worker bullying. The odds of experiencing WB by co-workers appeared to decline with age (OR: 0.96, 95% CI: 0.94–0.98), while occupations in construction, architecture, surveying and technical building services exhibit significantly higher odds (OR: 2.38, 95% CI: 1.31–4.31). WB by co-workers showed no associations with education, gender, professional status or geographical region. However, experiencing WB by co-workers was strongly associated with also experiencing WB by supervisors (OR: 19.21, 95% CI: 11.38–32.42).

Regarding the multivariable associations with WB by supervisors, age, again, demonstrated a significant association with exposure to bullying, with older employees being less likely affected (OR: 0.97, 95% CI: 0.95–0.99). Furthermore, the odds of experiencing WB by supervisors was lower for the region of East Germany (OR: 0.59, 95% CI: 0.39–0.88) compared to the average risk across all regions in Germany. Neither gender, education nor professional status were significantly associated with WB by supervisors. Yet, experiencing WB by supervisors was strongly associated with WB by co-workers (OR: 18.97, 95% CI: 11.28–31.88).

Multivariable analyses of WB were conducted again by including two-way interaction effects for age by gender, age by professional status, age by occupational area, gender by professional status, and gender by occupational area, respectively. The interaction between age and occupational area was significantly associated with overall WB (χ² = 21.61, *p* = 0.005) and WB by co-workers (χ² = 19.38, *p* = 0.013). The strongest age-related effect was observed among individuals in occupations in construction, architecture, surveying and technical building services. While younger adults are generally more affected by WB, this vulnerability is considerably more pronounced in those occupations for overall WB and WB by co-workers.

## Discussion

This study aimed to assess the prevalence of WB and to analyze its associations distinguished by the source of bullying. In total, 6.5% of the sample experienced WB. Younger age was consistently associated with a higher likelihood of reporting bullying overall, by co-workers, and by supervisors. In addition, the occupational area of construction, architecture, surveying and technical building services was significantly linked to overall WB and WB by co-workers. Further, overall WB, whether by supervisors or co-workers, was linked to female gender and blue-collar employment. Moreover, WB by supervisors and overall WB were less likely in East Germany, whereas no regional difference was found for co-worker bullying. Finally, there was a strong link between WB by supervisors and WB by co-workers, with individuals targeted by one group being more likely to also experience bullying from the other.

Prior research examining WB within cohorts of the German working population yielded prevalence estimates of similar magnitude [9, 8]. Results from the Study on Mental Health at Work, a representative panel study including employed individuals between the ages of 31 and 60, show a prevalence of WB of 6.7% [9]. Internationally, prevalences of WB have been shown to vary considerably [6, 3, 28, 4, 29]. Variations in prevalences can partly be explained by methodological differences in how bullying is assessed and operationalized. Nielsen et al. [29] examined variations in the prevalence of WB as a function of different assessment approaches. They report that estimates were highest for the self-labelling approach without a prior definition (18.1%) and lowest for self-labelling with a prior definition (11.3%) [29]. To provide potentially more robust estimates of WB, the present study employed a combined approach that integrates elements of self-labeling (feeling harassed) and behavioral experiences (unfair criticism, public embarrassment). In addition, differences in sample characteristics, changes in the awareness to WB in recent years, or cross-national differences in social protection systems (e.g., job security) may be potential explanatory factors contributing to variations in the observed prevalence estimates across studies [30].

In the current study, age emerged as a consistent factor associated with WB across different sources. Adjusting for covariates, younger employed individuals reported WB more likely than their older counterparts, regardless of whether the source of WB was regarded a supervisor or a co-worker. This is generally in line with previous studies showing younger individuals more frequently subjected to WB or hostile behaviors in the work environment [8, 31, 32]. Given that these studies date back over twenty years, it appears that the age effect of WB is not just a cohort effect. However, some studies have found divergent or less consistent results regarding younger age and WB. For instance, some studies could not find an age effect at all [3] or vice versa showed older employees more frequently subjected to WB than younger individuals [33]. In the literature, WB is commonly conceptualized as a phenomenon linked to power asymmetries or the misuse of authority [2, 33]. Accordingly, younger employees may be particularly vulnerable to WB, partly because they are more often situated in lower hierarchical roles and possess less work experience compared to older employees. Thus, variations between age and WB may be linked to the extent of hierarchical organization within companies, occupations or work groups.

Furthermore, female gender was associated with overall WB, with women having 51% higher odds of reporting overall WB than men. A comparable gender pattern emerged when WB by co-workers and WB by supervisors was analyzed separately, albeit not reaching statistical significance. Previous studies have provided heterogenous results regarding gender and WB. Several studies have found female employees more likely to be victims of WB or workplace harassment [34, 8, 35]. Others could not replicate such a gender effect [33, 3, 9]. Previous findings indicate that the relationship between gender and WB may vary depending on the occupation and sample analyzed. For instance, it has been suggested that groups with visible or distinct attributes that are different from the majority face an elevated risk of social exclusion or hostility [30, 36]. This may help to explain why men working in female-dominated occupations (e.g., nursing) might also be at increased risk of WB [37]. Further studies have reported nuanced patterns of association between gender and occupation, indicating a context-dependent link potentially influenced by additional factors, such as the type of leadership [30, 37]. In the present study we could not find an interaction of gender with either professional status or occupational area. Limited statistical power could be a possible explanation for our null result. In general, the link between gender and WB appears to be complex and multifaceted. Therefore, more studies are needed in the future investigating the specific contributions of gender to WB while taking occupational areas, workplace environments and leadership styles into account.

Overall WB was also more likely in blue-collar workers. Previous studies have repeatedly shown that individuals with low qualifications, temporary work contracts and those in blue-collar positions are at heightened risk of experiencing WB [3, 13, 9]. As with the effect of age, this is often linked to the low hierarchical positions, leaving employees in these positions more prone to the misuse of power by individuals in higher positions [9]. However, once WB was analyzed separately by source, the relationship between professional status and WB resembled the overall pattern only for WB by co-workers, although statistical estimates were imprecise. These results indicate that other aspects might be more strongly connected to WB, such as younger age or being already targeted by either supervisors or co-workers.

Furthermore, the occupational area of construction, architecture, surveying and technical building services had a more than two times higher likelihood to experience overall WB and WB by co-workers than other areas. Research on WB according to occupational groups or economic sector has produced mixed results. Overall, an increased risk of WB has been observed in several public sector occupations, such as health care, education, social services or administrative roles [3, 8, 30]. Job security and limited opportunities for transitioning into the private sector have been partly attributed to an increased risk of WB in occupations of the public sector [30]. In contrast, a higher potential for employee mobility, as found in hospitality and services, have been suggested to mitigate the endurance of hostile and bullying behaviors [30]. In addition, differences in work settings, in particular the absence of personal space at work, might be a relevant factor to consider. A recent study among the Swedish workforce reported an elevated risk of WB among employees working in open office environments compared to employees working in private or small shared offices [38]. Further, previous research has linked the perception of crowding with negative workplace behaviors among employees, with feelings of privacy invasion by supervisors or peers serving as a mediator of this relationship [39]. Thus, occupations with work settings that offer low levels of privacy may be at an increased risk for exposure to WB. Variations in results across occupational areas might indicate that different organizational structures are more relevant when analyzing factors associated with WB, for instance, role ambiguity, competition, low job autonomy and poor leadership styles [33, 40]. Furthermore, characteristics of the work environment and the organizational culture need to be considered in relation to the occurrence and duration of WB. Previous studies have shown a link between WB and performance-oriented, assertive or hostile work cultures [41, 42]. It has therefore been proposed that WB may serve as an indicator of the overall well-being of organizations [7].

Finally, we found that the occurrence of WB by supervisors appears to be different across regions in Germany. In comparison to the overall regional average in Germany, employees from East Germany reported significantly less WB by supervisors. No such regional differences were found for WB by co-workers. We can only hypothesize about the reason for this result. Due to its political and historical legacy, East Germany still differs from other regions in Germany with respect to available socioeconomic resources (e.g., individuals born in East Germany remain underrepresented in leadership positions [43]). Whether and how this translates into differences in supervisor behavior and differences between the frequency of WB between East Germany and other regions of the country remains to be investigated.

## Limitations

This study should also be considered in light of several limitations. The telephone survey achieved a response rate of 19.1%, which is comparable or higher to rates reported in previous telephone surveys [44–46]. Still, a potential selection bias should be considered, as certain marginalized groups of employees might have been less likely to participate (e.g. individuals with limited language proficiency), potentially limiting the generalizability of results. Secondly, the results are based on self-reported measures, which reflect the respondents’ perceptions and may be influenced by individual biases in self-assessment. Behavioral Instruments such as the LIPT questionnaire [47] measure exposure to, and the frequency of, predefined negative workplace behaviors, thereby offering a more standardized approach to assessing WB. However, these instruments have been criticized as well, because the behavioral experiencing approach relies exclusively on the researcher’s understanding of WB [30]. WB is fundamentally grounded in the subjective experience of the targeted individuals, and its work- and health-related consequences are largely determined by how these individuals perceive and interpret such behaviors. Thus, employing an approach that integrates self-labeling with behavioral experience, as in the present study, may produce more robust estimates of WB. A further limitation concerns the operationalization of occupational area. Participants were asked to classify the sector in which their employing company/organization operated, using response categories based on the occupational classification of the German Federal Employment Agency (KldB). For individuals who selected “other,” classification was based on occupation-related information (such as current occupation, a detailed description of duties, and alternative job titles). Therefore, the measure used in the present study represents a hybrid operationalization of sector and occupation rather than a uniform assessment of either construct. This approach was used for pragmatic and analytical reasons, because the classification of economic sectors would have resulted in a large number of response categories, while an exclusive use of the KldB variable resulted in insufficient cell sizes for some occupational categories. Thus, this approach may have introduced some degree of misclassification and needs to be considered when interpreting the results. Another limitation relates to the handling of missing data. Individuals with missing data on relevant variables were excluded casewise. Nonresponse analysis showed that excluded individuals differed from those included with regard to the level of education. As previous studies have indicated that employees with a lower education are at higher risk for experiencing WB, the findings of the present study may underestimate the true prevalence of WB, given that excluded individuals were more likely to have lower levels of education. Finally, as the analyses rely on cross-sectional data, the findings do not allow for inferences about the causal direction of the observed relationships.

## Conclusion

The present study highlights that, most notably, young employees are at heightened risk of experiencing bullying in the workplace, irrespective of whether the perceived source is a co-worker or a supervisor. Young individuals entering the workforce often start in low positions, possess limited work experience, and may therefore be more frequently exposed to hostile behaviors at work. The strong co-occurrence of bullying by co-workers and supervisors underscores the systemic nature of this phenomenon. The results of this study illustrate the complex and multifaceted nature of WB.

## Supplementary Information

Below is the link to the electronic supplementary material.

Supplementary Material 1

## Data Availability

The data generated and analyzed during this study is published as a Scientific Use File and can be accessed through the Research Data Centre of the Federal Institute for Occupational Safety and Health (FDZ-BAuA, https://www.baua.de/EN/Research/Research-Data/Workplace-bullying-study).

## Abbreviations

AAPOR: American Association for Public Opinion Research
BAuA: German Federal Institute for Occupational Safety and Health
BMAS: German Federal Ministry of Labour and Social Affairs
CASMIN: Comparative Analysis of Social Mobility in Industrial Nations
CI: Confidence interval
LIPT: Leymann Inventory of Psychological Terror
M: Mean
OR: Odds ratio
SD: Standard deviation
WB: Workplace bullying
