# Supplementary material for: Workplace bullying in a German representative sample of employees: a cross-sectional analysis
Source: J Occup Med Toxicol. 2026 Oct 21;21:37. doi: 10.1186/s12995-026-00535-3 (PMC13689300; doi:10.1186/s12995-026-00535-3)
Supplement: Supplementary file 1 [file 12995_2026_535_MOESM1_ESM.docx]

| **Table S1.** Prevalences of workplace bullying (WB) by source for individuals with ≥25 working hours per week. | | | | | | | | | |
| --- | --- | --- | --- | --- | --- | --- | --- | --- | --- |
|  | WB by supervisors/co-workers | | | WB by co-workers | | | WB by supervisors | | |
|  | n/N | % | [95%CI] | n/N | % | [95%CI] | n/N | % | 95%CI |
| **total** | 241/4,311 | 6.6 | [5.7-7.8] | 164/4,261 | 4.6 | [3.8-5.7] | 140/4,236 | 3.7 | [2.9-4.5] |
| **Age** |  |  |  |  |  |  |  |  |  |
| 18-29 | 42/312 | 11.8 | [8.5-16.1] | 31/312 | 8.3 | [5.6-12.0] | 23/305 | 6.2 | [3.9-9.6] |
| 30-39 | 51/648 | 7.0 | [5.2-9.3] | 35/641 | 4.9 | [3.5-7.0] | 27/637 | 3.6 | [2.4-5.4] |
| 40-49 | 65/921 | 6.9 | [5.3-9.0] | 47/917 | 4.9 | [3.6-6.7] | 38/910 | 4.1 | [2.8-5.8] |
| 50-59 | 53/1,664 | 3.1 | [2.3-4.2] | 32/1,644 | 1.9 | [1.3-2.9] | 33/1,635 | 1.9 | [1.3-2.7] |
| 60+ | 30/766 | 3.6 | [2.4-5.2] | 19/747 | 2.2 | [1.3-3.6] | 19/749 | 2.2 | [1.3-3.5] |
| **gender** |  |  |  |  |  |  |  |  |  |
| male | 112/2,237 | 5.5 | [4.4-6.8] | 74/2,216 | 3.7 | [2.8-4.8] | 65/2,196 | 3.1 | [2.3-4.1] |
| female | 129/2,074 | 8.1 | [6.5-9.9] | 90/2,045 | 5.7 | [4.4-7.4] | 75/2,040 | 4.4 | [3.3-5.9] |
| **education^a^** |  |  |  |  |  |  |  |  |  |
| high | 93/1,900 | 5.7 | [4.5-7.3] | 61/1,885 | 4.0 | [2.9-5.4] | 53/1,850 | 3.1 | [2.3-4.3] |
| middle | 126/2,071 | 7.3 | [5.9-9.1] | 82/2,046 | 4.8 | [3.6-6.3] | 76/2,047 | 4.2 | [3.1-5.6] |
| low | 22/340 | 6.4 | [3.9-10.4] | 21/330 | 6.4 | [3.8-10.5] | 11/339 | 2.8 | [1.4-5.6] |
| **region** |  |  |  |  |  |  |  |  |  |
| north | 42/723 | 7.3 | [4.9-10.7] | 28/711 | 4.7 | [2.9-7.6] | 27/707 | 4.5 | [2.7-7.2] |
| west | 73/1,405 | 6.1 | [4.6-7.9] | 50/1,389 | 4.6 | [3.3-6.5] | 44/1,381 | 3.3 | [2.3-4.8] |
| south | 84/1,252 | 8.3 | [6.4-10.6] | 57/1,240 | 5.2 | [3.7-7.0] | 43/1,232 | 4.6 | [3.2-6.5] |
| east | 42/931 | 4.6 | [3.2-6.6] | 29/921 | 3.7 | [2.4-5.7] | 26/916 | 2.2 | [1.4-3.4] |
| **professional status** |  |  |  |  |  |  |  |  |  |
| apprentice | 8/46 | 14.0 | [6.6-27.4] | 6/46 | 10.5 | [4.4-22.7] | 3/45 | 5.1 | [1.4-17.1] |
| salaried employee | 185/3,496 | 5.9 | [5.0-7.1] | 124/3,452 | 3.9 | [3.2-4.8] | 110/3,435 | 3.6 | [2.8-4.5] |
| civil servant | 14/361 | 4.3 | [2.2-8.2] | 10/359 | 2.9 | [1.4-6.1] | 9/353 | 3.1 | [1.3-7.0] |
| blue-collar worker | 34/408 | 9.9 | [6.6-14.5] | 24/404 | 7.9 | [4.9-12.4] | 18/403 | 3.8 | [2.2-6.4] |
| **occupational area** |  |  |  |  |  |  |  |  |  |
| occupations in agriculture, forestry, farming, and landscaping | 3/70 | 1.7 | [0.5-5.9] | 1/68 | 1.1 | [0.2-5.5] | 3/69 | 1.7 | [0.5-6.0] |
| occupations in production of raw materials and goods, and manufacturing | 36/648 | 6.8 | [4.6-9.8] | 24/647 | 3.9 | [2.5-6.2] | 22/642 | 4.4 | [2.6-7.1] |
| occupations in construction, architecture, surveying and technical building services | 28/348 | 10.7 | [6.8-16.2] | 21/342 | 8.6 | [5.1-13.9] | 14/342 | 4.5 | [2.5-8.1] |
| occupations in natural sciences, geography and informatics | 11/284 | 2.8 | [1.4-5.5] | 7/282 | 1.4 | [0.6-3.3] | 8/80 | 2.3 | [1.0-5.0] |
| occupations in transit, logistics, safety and security, and military | 30/387 | 8.8 | [5.7-13.2] | 20/383 | 6.4 | [3.8-10.5] | 18/377 | 5.2 | [3.0-8.8] |
| occupations in commercial services, trading, sales, the hotel business and tourism | 34/619 | 6.9 | [4.6-10.4] | 21/613 | 4.9 | [2.9-8.2] | 22/609 | 3.9 | [2.2-6.8] |
| occupations in business organization, accounting, law and administration | 19/556 | 4.2 | [2.5-7.0] | 12/552 | 2.7 | [1.4-5.2] | 14/549 | 2.9 | [1.6-5.5] |
| occupations in health care, the social sector, teaching and education | 71/1,214 | 6.9 | [5.1-9.4] | 52/1,192 | 5.1 | [3.5-7.2] | 34/1,189 | 3.2 | [2.0-5.0] |
| occupations in philology, literature, humanities, social sciences, economics, media, art, culture, and design | 9/185 | 4.9 | [2.3-10.1] | 5/182 | 2.8 | [1.0-7.8] | 5/179 | 2.9 | [1.1-7.2] |
| Note: WB = workplace bullying; N = unweighted total sample size; n = unweighted sample size of subgroups affected by WB; % = weighted percentage; SD = standard deviation; CI = confidence interval; ^a^classification according to the international new CASMIN educational classification (24). | | | | | | | | | |
